# Sexing of cattle embryos using RNA-sequencing data or polymerase chain reaction based on a complete sequence of cattle chromosome Y

**DOI:** 10.3389/fgene.2023.1038291

**Published:** 2023-04-03

**Authors:** Jada Lindsay Nix, Gustavo Pimenta Schettini, Fernando Henrique Biase

**Affiliations:** School of Animal Sciences, Virginia Polytechnic Institute and State University, Blacksburg, VA, United States

**Keywords:** sexing, RNA-sequencing, blastocyst, embryos, PCR

## Abstract

When necessary, RNA-sequencing data or polymerase chain reaction (PCR) assays can be used to determine the presence of the chromosome Y (ChrY) in samples. This information allows for biological variation due to sexual dimorphism to be studied. A prime example is when researchers conduct RNA-sequencing of single embryos, or conceptuses, prior to the development of gonads. A recent publication of a complete sequence of the ChrY has removed limitations for the development of these procedures in cattle, otherwise imposed by the absence of a ChrY in the reference genome. Using the sequence of the cattle ChrY and transcriptome data, we conducted a systematic search for genes in the ChrY that are exclusively expressed in male tissues. The genes ENSBIXG00000029763, ENSBIXG00000029774, ENSBIXG00000029788, and ENSBIXG00000029892 were consistently expressed across male tissues and lowly expressed or absent in female samples. We observed that the cumulative values of counts per million were 2688-fold greater in males than the equivalent values in female samples. Thus, we deemed these genes suitable for the sexing of samples using RNA-sequencing data. We successfully used this set of genes to infer the sex of 22 cattle blastocysts (8 females and 14 males). Additionally, the completed sequence of the cattle ChrY has segments in the male-specific region that are not repeated. We designed a pair of oligonucleotides that targets one of these non-repeated regions in the male-specific sequence of the ChrY. Using this pair of oligonucleotides, in a multiplexed PCR assay with oligonucleotides that anneal to an autosome chromosome, we accurately identified the sex of cattle blastocysts. We developed efficient procedures for the sexing of samples in cattle using either transcriptome data or their DNA. The procedures using RNA-sequencing will greatly benefit researchers who work with samples limited in cell numbers which are only sufficient to produce transcriptome data. The oligonucleotides used for the accurate sexing of samples using PCR are transferable to other cattle tissue samples.

## Introduction

Determining sexual dimorphism has become increasingly important in biomedical studies (Clayton, 2018). Determining the sex of a sample collected from a mammalian individual before the development of gonads (for example, in pre-implantation embryos) requires the detection of DNA or RNA originating from the chromosome Y. Notably, however, the sequence of the cattle chromosome Y has not yet been added to the reference genome. This poses challenges for the identification of male specific sequences or genes for the development of assays or procedures to infer the sex of embryos or samples of unknown origin.

Non-reproductive tissues express genes from the chromosome Y (Chang et al., 2013; Zhang et al., 2019; Godfrey et al., 2020). Thus, the chromosome Y has been used as a potential source of transcripts for sexing samples of unknown origin. For example, transcriptome data has been used for the determination of sex in humans (Petropoulos et al., 2016), rhesus macaque (Midic et al., 2018), and mice (Midic et al., 2018) pre-implantation embryos. In addition, transcriptome data was used to determine the sex of pig conceptuses (Teixeira et al., 2019). A major difference between these species and cattle is that the chromosome Y is not yet included in the reference genome. An opportunity to identify the sex of samples using RNA-sequencing data emerged with the recent publication (Liu et al., 2019) of the complete sequence of the cattle chromosome Y, accompanied by annotation of this chromosome.

In cattle, sex determination of preimplantation embryos by polymerase chain reaction (PCR) (Mullis, 1990) of male-specific genomic regions has been successfully employed in previous studies (Bondioli et al., 1989; Peura et al., 1991; Utsumi et al., 1992; Itagaki et al., 1993; Kirkpatrick and Monson, 1993; Machaty et al., 1993; Bredbacka et al., 1995; Park et al., 2001; Alves et al., 2003; Hirayama et al., 2004; Kageyama et al., 2004; Ekici et al., 2006; Lu et al., 2007; Zoheir and Allam, 2010; Khamlor et al., 2015) with an average success rate of approximately 90% (Herr, 1990; Itagaki et al., 1993; Seidel, 2003). This method has also been successfully used in sexing animal products, such as semen (Joerg et al., 2004; Parati et al., 2006; Abdul et al., 2011; Resende et al., 2011; Maleki et al., 2013), blood (Fujishiro et al., 1995), or meat (TAGLIAVINI et al., 1993; Zeleny et al., 2002; Herrero et al., 2013). In addition, maternal plasma (Da Cruz et al., 2012; Davoudi et al., 2012) and fetal fluids (Kamimura et al., 1997; Makondo et al., 1997) have undergone PCR for sexing of gestational fetuses. These studies, however, utilized oligonucleotide sequences which were designed based on an incomplete bovine chromosome Y sequence. Many of these oligonucleotides bind to repeated regions on the chromosome Y (Bondioli et al., 1989; Utsumi et al., 1992; Itagaki et al., 1993; Machaty et al., 1993; Bredbacka et al., 1995; Park et al., 2001; Alves et al., 2003; Kageyama et al., 2004). The amplification of repeated elements has been shown to negatively impact oligonucleotide binding specificity while increasing the instance of PCR artifacts, overall lowering assay efficiency and accuracy (Hommelsheim et al., 2014). In attempts to combat this downfall, previous studies have relied on methods such as nested PCR (Kirkpatrick and Monson, 1993; Makondo et al., 1997; Abdul et al., 2011) or loop-mediated isothermal amplification (Hirayama et al., 2004; Zoheir and Allam, 2010; Khamlor et al., 2015) to improve assay accuracy and sensitivity. However, replicability of the assays across laboratories has been challenging.

The recent publication of the complete sequence of the cattle chromosome Y (Liu et al., 2019), along with a gene annotation, motivated us to develop a procedure to determine sample sex using cattle RNA-sequencing data. First, we aimed to identify a set of genes in the chromosome Y that can be used for the determination of sex using transcriptome data. Next, we tested the hypothesis that RNA-sequencing data can be used for accurate determination of sex of cattle embryos. In parallel, we designed a new set of oligonucleotides that can accurately detect DNA from the chromosome Y in samples of unknown sex. Using transcriptome data from blastocysts as a testbed, we present a pipeline for the unambiguous determination of sex in cattle samples.

## Materials and methods

All codes used for bioinformatics and analytical work related to RNA-sequencing reported in the methods are presented in the Additional file 1, and are also available in the figshare repository (Biase, 2022) and on the website: https://biase-lab.github.io/sexing_samples_RNA_sequencing/index.html.

### Public datasets used for the identification of genes suitable for sexing by RNA-sequencing

We used public RNA-sequencing datasets produced from samples obtained from male or female cattle. Datasets from females [GSE55435 (Canovas et al., 2014), GSE192530 (Wilson et al., 2022)] contained samples from the endometrium, fat, hypothalamus, liver, muscle, ovary, pituitary, peripheral white blood cells, and uterus. Datasets from males [GSE176219 (Rabaglino et al., 2021), GSE196974 (Rabaglino et al., 2021), GSE128075 (Fang et al., 2020)] contained epididymis, liver, muscle, prostate, and testis.

### Identification of genes in the cattle chromosome Y suitable for sexing with RNA-sequencing data

We obtained the sequence for the cattle chromosome Y from the National Center for Biotechnology Information [CM011803.1, assembly GCA_003369685.2 produced by Liu et al. (2019)], and appended the sequence to the file containing the cattle reference genome (Elsik et al., 2009) (ARS-UCD1.2/bosTau9) obtained from the Ensembl database (Kinsella et al., 2011; Flicek et al., 2012), hereafter denoted as ARS-UCD1.2+chrY. Furthermore, we obtained the reference annotation for the cattle genome (Bos_taurus.ARS-UCD1.2.105.gtf) and the annotation from the GCA_003369685.2 assembly (Bos_taurus_hybrid.UOA_Angus_1.97.chr.gtf) from the Ensembl database (Kinsella et al., 2011; Flicek et al., 2012). Because we were interested in the genes specific to the chromosome Y, we only worked with the annotation ranging from nucleotides 6953027 and 15591347 (Liu et al., 2019).

We aligned the RNA-sequencing data to the genome ARS-UCD1.2+chrY using HISAT2 (2.2.1) (Kim et al., 2019), followed by filtering with samtools (Li et al., 2009; Danecek et al., 2021) and removal of duplicates with biobambam (Tischler and Leonard, 2014). Next, we counted the sequences matching the reference annotation (Bos_taurus.ARS-UCD1.2.105.gtf) and the modified annotation from Liu et al. (Liu et al., 2019). We executed the counting independently to avoid merging GTF files.

In R software (Ihaka and Gentleman, 1996; RCoreTeam, 2020), we created one matrix with the read counts from both annotations and used it as input to calculate normalized counts per million with the functions “calcNormFactors” (TMM normalization) and “cpm” from the R package “edgeR” (McCarthy and Smyth, 2010). Next, we filtered the genes present in the chromosome Y to retain genes with more than 600 reads across 64 male and female samples. Finally, the raw counts from genes present in the chromosome Y were used as input for the calculation of differential gene expression using the quasi-likelihood negative binomial generalized log-linear model from the R package “edgeR.”

### Production of RNA-sequencing data from blastocysts

Reagents used for embryo production were purchased from Sigma-Aldrich (St. Louis, MO, United States), unless otherwise stated.

We obtained cattle ovaries from a slaughterhouse which were transported to our laboratory in saline (0.9% NaCl) and antibiotic antimycotic solution (1x, Life Technologies, Grand Island, NY, United States). Using a regulated vacuum system coupled to a collection tube containing BoviPlus oocyte washing medium with BSA (Minitube, Verona, WI, United States) (OCM) and an 18 g needle, we aspirated ovarian follicles ranging from 3 to 8 mm in diameter to retrieve cumulus-oocyte complexes (COCs). COCs were then placed through two consecutive washes in OCM. Only COCs with heterogenous cytoplasm and three or more layers of compact cumulus cells surrounding the zona pellucida were *in vitro* matured.

All procedures and media formulations were adapted from a standard protocol for *in vitro* production of cattle embryos (Tribulo et al., 2019). The COCs were first placed through three washes of oocyte maturation media [OMM, TCM-199 + Earle’s Salts (Life Technologies, Grand Island, NY, United States) supplemented with 5 μg/ml follicle-stimulating hormone (Vetoquinol, Ft. Worth, TX, United States), 46 µM gentamycin, 1 mM glutamax, 250 mM sodium pyruvate, and 10% FBS (HyClone Laboratories, Utah, United States)]. This was followed by plating ten COCs in 50 µl droplets of OMM covered in mineral oil, which were incubated at 38.5°C in a humidified atmosphere at 5% CO_2_ for 22–24 h.

Prior to fertilization, the COCs were washed trice in SOF-HEPES (4-(2-hydroxyethyl)-1-piperazineethanesulfonic acid)-buffered synthetic oviductal fluid (3 mg/ml fraction V BSA, 0.2 mM sodium pyruvate, 7.5 μg/ml gentamicin, 5.3 mM sodium-lactate, 107.7 mM sodium chloride, 10 mM HEPES, 2 mM sodium bicarbonate, 1.17 mM calcium chloride dihydrate, 1.19 mM potassium phosphate monobasic, 7.16 mM potassium chloride, and 0.49 mM magnesium chloride hexahydrate) and twice in fertilization media (6 mg/ml essentially fatty acid free BSA, 1 mM sodium pyruvate, 10.5 mM gentamicin, 0.01 mg/ml heparin, 1 mM caffeine, 5.3 mM sodium-lactate, 107.7 mM sodium chloride, 25.07 mM sodium bicarbonate, 1.17 mM calcium chloride dihydrate, 1.19 mM potassium phosphate monobasic, 7.16 mM potassium chloride, and 0.49 mM magnesium chloride hexahydrate) before being placed in a final plate of fertilization media. In parallel, straws containing frozen semen were prepared following a previously described protocol (Tribulo et al., 2019) with BoviPure and BoviDilute (Nidacon International, Molndal, Sweden) to separate spermatozoa by a density isolation gradient. Spermatozoa were introduced to COCs in our final fertilization plate at a concentration of 1,000,000 spermatozoa/ml, as described elsewhere (Ward et al., 2002). Plates containing COCs and spermatozoa were incubated for 16–18 h under the same conditions described for IVM.

Presumptive zygotes were denuded of their cumulus cells by vortexing for 5–7 min, then transferred through three washes of SOF-HEPES. Presumptive zygotes were then placed through two washes of SOF culture media (4 mg/ml essentially fatty acid free BSA, 5.3 mM sodium-lactate, 107.7 mM sodium chloride, 25.07 mM sodium bicarbonate, 1.17 mM calcium chloride dihydrate, 1.19 mM potassium phosphate monobasic, 7.16 mM potassium chloride, and 0.49 mM magnesium chloride hexahydrate, 1 mM glutamax, 0.4 mM sodium pyruvate, 1x MEM-non-essential amino acid solution, 1x BME-essential amino acid solution, 52.3 mM gentamicin, 0.5 mM sodium citrate, and 2.77 mM myo-inositol) before being plated in groups of 25–30 in 50 μl droplets of SOF culture media immersed in mineral oil. Plates containing presumptive zygotes in SOF culture media were incubated for 7 days at 38.5°C in a humidified atmosphere at 5% CO_2_ and 5% O_2_. We collected whole blastocysts individually in 1 μl of phosphate buffered saline solution, between 165 and 166 h post-fertilization (hpf).

### Sexing of blastocysts using polymerase chain reaction

Using NCBI Primer BLAST (Ye et al., 2012), oligonucleotides (F: 5′-AGG​GTG​AAG​CAA​ATG​GTC​GT-3′, R: 5′-GGA​GCA​ACA​GTG​TCC​TGT​GT-3′) were designed to target the male-specific region of the Y (MSY) chromosome [CM011803.1 (Liu et al., 2019)]. These oligos produce a 279 nucleotide-long amplicon starting at the nucleotide 10308598 and ending at the nucleotide 10308776 of the sequence CM011803.1. Oligonucleotides targeting a portion of the gene CDK1 (F: 5′-GCC​CAG​ACC​CAG​CAT​CAT​T-3′, R: 5′-GGG​AGT​GCC​CAA​AGC​TCT​AAA-3′), located on chromosome 28, were multiplexed in addition to those which targeted MSY. These oligonucleotides were designed to produce 590 nucleotide-long amplicons starting at the nucleotide 16498587 and ending at the nucleotide 16499156 of chromosome 28.

A total of 60 bovine blastocysts were collected individually in 1 μl phosphate buffered saline solution. The embryo’s DNA was exposed by the addition of 5 μl of Lucigen QuickExtract DNA Extraction Solution (Lucigen, Middleton, WI, United States) per embryo prior to incubation at 65°C for 15 min then 98°C for 2 min.

We obtained post-mortem blood samples from a heifer and a steer. We isolated the buffy coat by centrifugation as we have previously described (Dickinson et al., 2018; Moorey et al., 2020; Wilson et al., 2022). Using a Zymo Direct-zol DNA/RNA Miniprep Kit (Zymo Research, Irvine, CA, United States), we extracted DNA from the isolated peripheral white blood cells to serve as positive controls.

Using the total lysate of each blastocyst, we conducted PCR reactions comprised of 0.2 IU/μl Phusion Hot Start II DNA Polymerase (Thermofisher Scientific, Waltham, MA, United States), 1X Phusion HF Buffer, 3% DMSO, 200 μM dNTPs (Promega, Madison, WI, United States), and forward and reverse oligonucleotides (IDT, Coralville, Iowa) for CDK1 and MSY at 0.15 μM each, at a final volume of 20 μl in clear 0.2 ml PCR tubes (VWR, Philadelphia, PA, United States). The cycling conditions used were as follows: 98°C for 30 s, then 35 cycles of 98°C for 10 s, 60°C for 30 s, and 72°C for 30 s, followed by a final extension of 72°C for 5 min.

We determined the sex of each embryo by agarose gel electrophoresis. We loaded 10 μl of each PCR product in a 2% agarose gel (VWR, Philadelphia, PA, United States) in 1x Tris-Acetate EDTE buffer (Thermofisher, Waltham, MA, United States) and electrophoresis was carried out for 40 min at 90v. DNA was stained by submerging the gel into a solution of Promega Diamond Nucleic Acid Dye (Promega, Madison, WI, United States) and 1x Tris-Acetate EDTE buffer for 30 min away from direct light. Using a BioRad Gel Doc XR and Image Lab program (BioRad, Hercules, CA, United States), gels were imaged. Males were defined as those who presented bands from both CDK1 and MSY amplification, while females were defined as those who only presented a band for CDK1 amplification.

To validate the identity of our amplicon, we proceeded with Sanger sequencing of PCR products resultant from MSY chromosome oligonucleotides. We conducted PCR on known male DNA extracted from peripheral white blood cells, using reaction and cycling conditions as described above. We then purified amplicon fragments with a Zymo DNA Clean and Concentrator kit (Zymo Research, Irvine, CA, United States) by following the provided protocol. We quantified 2 μl of our purified product using a Nanodrop (Thermofisher Scientific, Waltham, MA, United States). We submitted the purified fragment for Sanger sequencing by the Virginia Tech Genomic Sequencing Center. Resultant sequences were aligned to a segment of the *Bos indicus* x *Bos taurus* breed Angus x Brahman F1 hybrid chromosome Y whole genome shotgun sequence (CM011803.1) to validate amplicon identity using BLAST from the NCBI (Johnson et al., 2008; Boratyn et al., 2013).

### Sexing of blastocysts using RNA-sequencing data

We extracted total RNA from individual blastocysts with TRIzol Reagent with the aid of Phasemaker Tubes for improved yield and purity of the RNA (Chomczynski and Sacchi, 1987; Puissant, 1990; Chomczynski and Sacchi, 2006; Biase, 2021), and stored total RNA in 70% ethanol at −80°C until further use. Before proceeding with library preparation, we assessed the RNA integrity resulting from our extraction in a 2100 Bioanalyzer using the Agilent RNA 6000 Pico Kit (Agilent Technologies, Germany). Samples with RNA integrity number greater than 8 were used for library preparation.

We carried out library preparation using a modified mcSCRB-seq protocol (Bagnoli et al., 2018; Biase, 2021). Ethanol was removed from the RNA pellets and dried for a few minutes. Next, we resuspended the RNA pellet in a solution containing an oligo-dTVN primer (5′-AAGCAGTGGTATCAACGCAGAGTACT30VN-3′). Tubes were heated to 72°C for 3 min to denature secondary RNA structures. Then, we added 5 µl of RT mix containing 200 U/µl Maxima H Minus Reverse Transcriptase, 1x Maxima RT Buffer, 7.5% PEG 8000, 10 mM dNTPs, and 2 µM of a template-switching oligo (5′-AAGCAGTGGTATCAACGCAGAGTACATrGrG+G-3′) to the reaction. We incubated the tubes at 42°C for 1.5 h to generate full-length cDNA. Next, we purified the cDNA product with AMPure XP beads to remove reagents before amplification.

Immediately following the purification, we added a preamplification mix containing 1.25 U Terra polymerase, 1x Terra direct buffer, and 0.1 µM of primer (5′-AAG​CAG​TGG​TAT​CAA​CGC​AGA​GT-3′) to each tube. Amplification was performed with 10 PCR cycles under the following conditions: 98°C for 15 min, 68°C for 5 min, and 72°C for 10 min. After the final cycle, the reaction was held at 8°C. Finally, we purified products with AMPure XP beads. We assessed the quality of the amplification with the 2100 Bioanalyzer and the Agilent High Sensitivity DNA kit, followed by quantification of the products with a Qubit 4.0 fluorometer.

We used 1 ng of the amplified cDNA as a template for library preparation using the Nextera DNA Flex Library Prep kit following the manufacturer’s protocol. We amplified the tagmented cDNA with 13 cycles of PCR. Following a purification of the library with AMPure XP beads, we assessed the DNA profile with a 2100 Bioanalyzer, using the Agilent High Sensitivity DNA kit and quantified with a Qubit 4 fluorometer. Libraries were sent to the VANTAGE: Vanderbilt Technologies for Advanced Genomics at Vanderbilt University to produce paired-end sequencing with reads 150 nucleotides long, using an Illumina NovaSeq6000 platform.

Using the pipeline described above, we aligned the sequences to the bovine reference genome and the chromosome Y (ARS-UCD1.2+chrY described above), followed by filtering and removal of duplicates before the quantification of genes in the autosome, X, and Y chromosomes.

## Results

### Identification of genes suitable for sexing with RNA-sequencing data

We obtained 30 and 36 libraries produced from samples collected from males [GSE176219 (Rabaglino et al., 2021), GSE196974 (Rabaglino et al., 2021), GSE128075 (Fang et al., 2020)] and females [GSE55435 (Canovas et al., 2014), GSE192530 (Wilson et al., 2022)], respectively, from the GEO public database. On average, after filtering, there were 48,700,745, and 17,829,956 fragments mapped to the reference cattle genome and chromosome Y for females and males, respectively. Out of the reads that mapped to the whole genome, on average, 0.14% and 0.1% mapped to chromosome Y in males and females, respectively.

After calculating the normalized counts per million using all the reads that mapped to the cattle annotation, we subset the transcriptome dataset to retain genes that span the window between nucleotides 6953027 and 15591347, which includes the ampliconic region of chromosome Y. After testing for differential transcript abundance between males and females, we identified a few candidate genes based on the F value obtained from the quasi-likelihood negative binomial generalized log-linear model executed with the R package “edgeR” (Table 1).

**TABLE 1:** Top ten differentially expressed genes between male and female tissues located in the cattle chromosome Y.

Gene ID	Gene symbol^a^	logFC	logCPM	F	P	ChrY start^b^	ChrY end^b^
ENSBIXG00000029763	*OFD1*	11.27	14.75	2118.43	7.92E−37	6953027	7017526
ENSBIXG00000029788	—	9.71	14.55	1046.01	3.99E−46	7419104	7602856
ENSBIXG00000029774	*ZRSR2*	10.98	14.47	813.87	1.34E−28	7305910	7361376
ENSBIXG00000029892	—	8.46	15.50	707.08	3.41E−40	8054318	8104065
ENSBIXG00000029986	—	9.59	13.15	429.26	2.75E−23	10463946	10554936
ENSBIXG00000029770	—	7.97	16.16	341.26	4.12E−29	7099749	7248718
ENSBIXG00000029886	—	−1.95	18.42	320.06	6.13E−29	7835303	7835773
ENSBIXG00000029889	—	6.60	15.14	228.70	5.33E−23	7966867	8040039
ENSBIXG00000029864	—	6.73	15.57	210.27	4.26E−22	7648102	7686255
ENSBIXG00000030021	—	−2.46	14.86	172.28	3.28E−21	11907972	11908877

^a^ Ensembl annotation. — not determined. FC, fold change, CPM, counts per million.

^b^ Please refer to (Bos_taurus_hybrid.UOA_Angus_1.97.chr.gtf) from the Ensembl database (Kinsella et al., 2011; Flicek et al., 2012).

**FIGURE 1:**
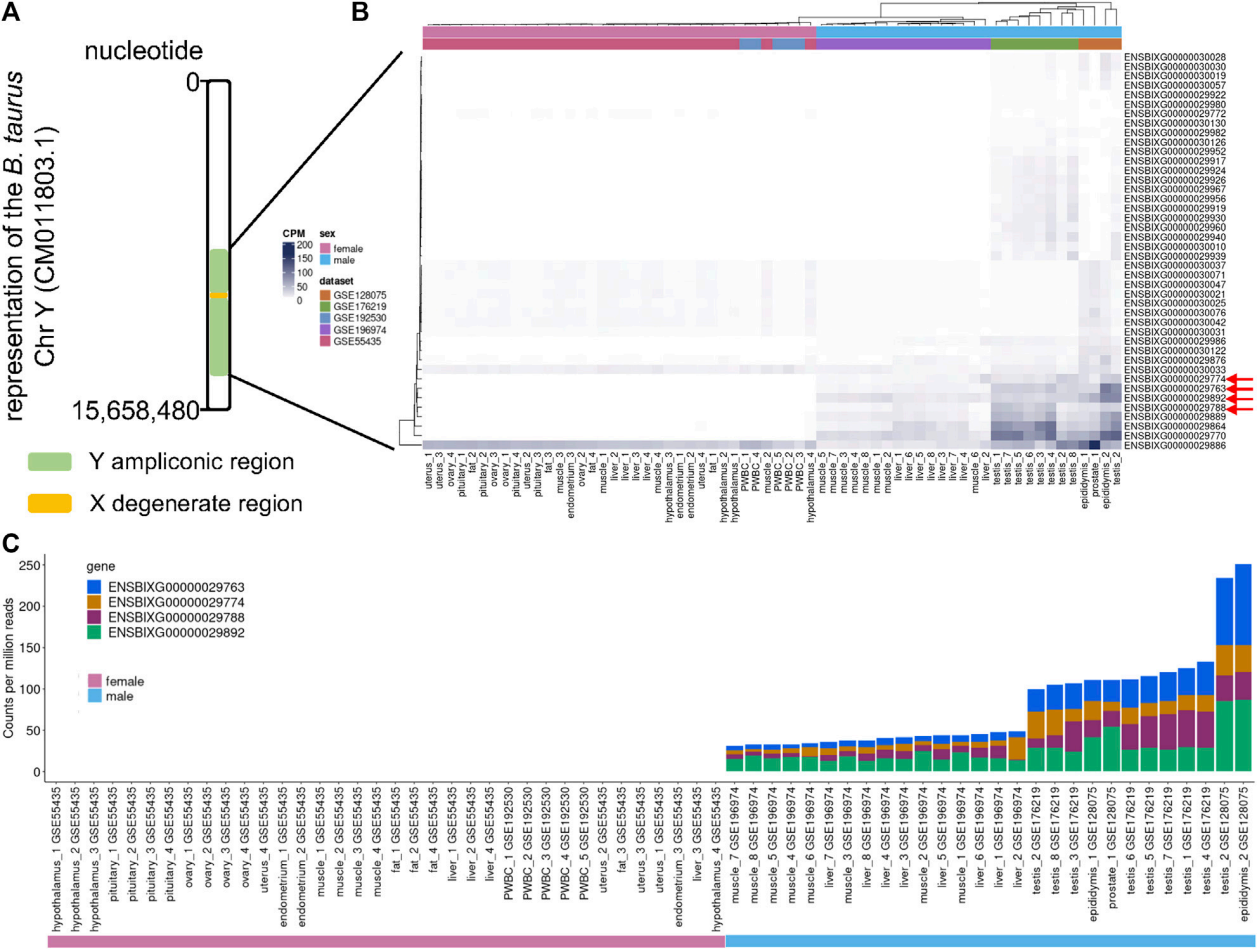
Using RNA-sequencing data to detect transcripts specific to male samples. **(A)** Depiction of the cattle chromosome Y. **(B)** Representation of the transcript abundance of genes annotated to the ampliconic region in the cattle chromosome Y. Red arrows indicate four candidate genes with transcripts enriched in tissue samples collected from males. **(C)** Cumulative counts per million reads of the genes ENSBIXG00000029788, ENSBIXG00000029892, ENSBIXG00000029774, and ENSBIXG00000029763 in samples obtained from male and female cattle.

We inspected the profile of the genes within the ampliconic region of the cattle chromosome Y (Figure 1A) and observed that the samples obtained from females had low to no transcript quantification for the top four differentially expressed genes (Figure 1B, red arrows). We identified that the cumulative transcript abundance (in counts per million, CPM) for the genes ENSBIXG00000029788, ENSBIXG00000029892, ENSBIXG00000029774, and ENSBIXG00000029763 in male samples was on average 2688-fold greater than the equivalent values in female samples (male samples minimum: 30.92, median: 46.76, average: 80.65, maximum: 250.85; female samples minimum: 0.00, median: 0.00, average: 0.03, maximum: 0.37) (Figure 1C).

### Sexing blastocysts by RNA-sequencing data

**FIGURE 2:**
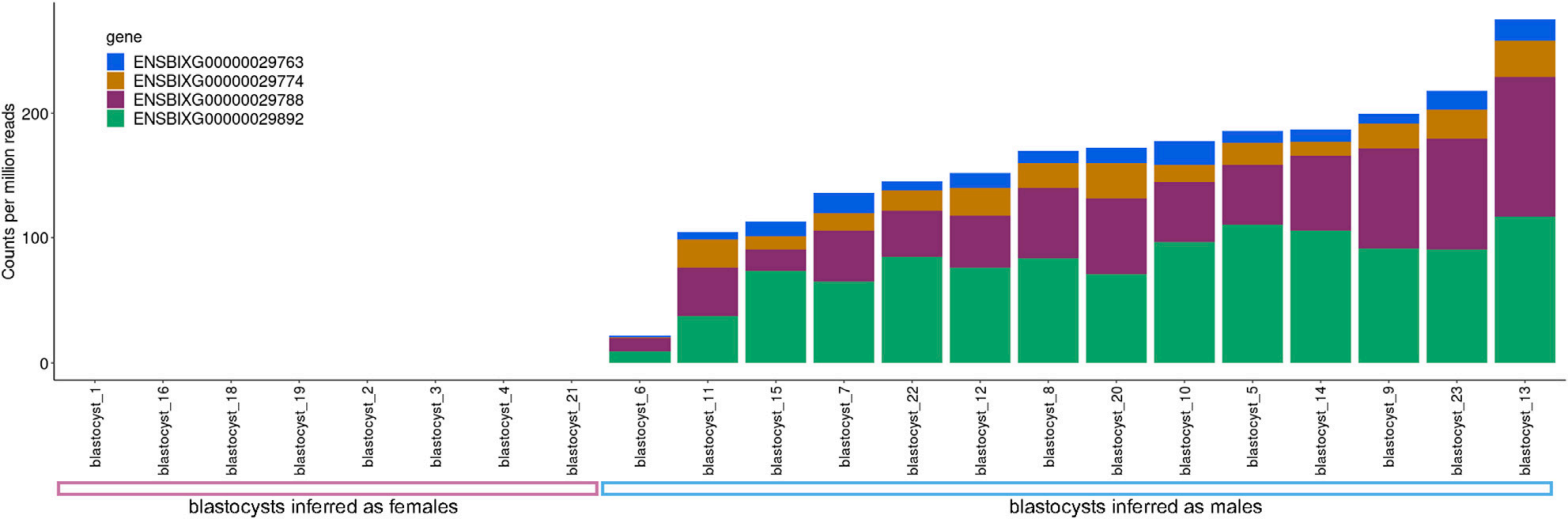
Sexing of cattle blastocysts using RNA-sequencing data.

We assessed the transcript abundance of the genes ENSBIXG00000029788, ENSBIXG00000029892, ENSBIXG00000029774, and ENSBIXG00000029763 in 22 cattle blastocysts. For eight blastocysts there was no detection of transcripts (CPM = 0) for the genes ENSBIXG00000029788, ENSBIXG00000029774, and ENSBIXG00000029763. For seven blastocysts there was no detection of transcripts (CPM = 0) for the gene ENSBIXG00000029892, whereas one blastocyst presented CPM = 0.0592 for ENSBIXG00000029892. The average cumulative CPM for ENSBIXG00000029788, ENSBIXG00000029892, ENSBIXG00000029774, and ENSBIXG00000029763 for these eight blastocysts was equal to zero, therefore, we inferred them to be females (Figure 2). By comparison, we observed a group of 14 blastocysts from which sequences from transcripts of the genes ENSBIXG00000029788, ENSBIXG00000029892, ENSBIXG00000029774, and ENSBIXG00000029763 were produced and quantified. Their average cumulative CPM was 161.38 (minimum: 22.02, median: 171.32, maximum: 275.28) and thus we inferred these blastocysts to be males.

### Sexing embryos by PCR

**FIGURE 3:**
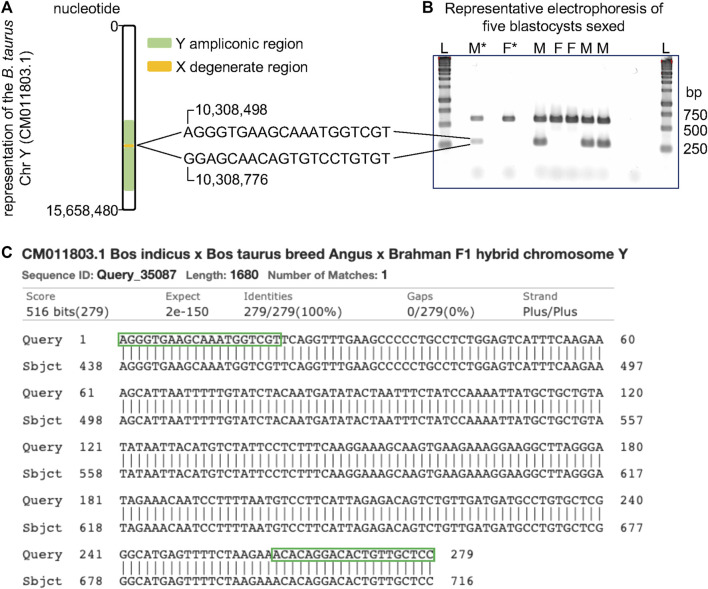
Schematics of PCR assay for sexing samples from cattle. **(A)** Position of the oligonucleotides relative to the sequence of the cattle chromosome Y (Liu et al., 2019). **(B)** Representative electrophoresis of the result of PCR assays for sexing cattle samples. **(C)** Alignment of the PCR product with a segment of the sequence of the cattle chromosome Y (CM011803.1). Green boxes indicate where the oligos align on the DNA sequence. L, ladder, F*, control DNA from a female, F, sample inferred as female, M*, control DNA from a male, M, sample inferred as male, bp, base pairs.

Using the complete sequence of the cattle chromosome Y, recently published (Liu et al., 2019), we identified a set of oligonucleotides spanning the nucleotides 10,308,490 and 10,308,776 that pair in the ampliconic region (Figure 3A). Because these oligonucleotides did not align in a repeated region of the cattle chromosome Y, the amplified fragment is expected to be 279 base pairs long. Indeed, our tests using male DNA showed that these oligonucleotides produced a fragment of length close to the expected (Figure 3B). More importantly, PCR assays using these two oligonucleotides did not produce amplicons when female DNA was used. We multiplexed this reaction with a pair of oligonucleotides mapping to the cattle chromosome 28, thus a reaction would have one product of amplification serving as a positive control. Therefore, using our oligonucleotides, one band indicates a female and two bands indicate a male DNA (Figure 3B). We further confirmed the amplicon by sequencing the fragment amplified using the Sanger method (Additional file 2). The amplified product aligned to the sequence of the chromosome Y deposited in the NCBI (Figure 3C) and did not map to the reference genome (data not shown). Using the described PCR assay with two pairs of oligonucleotides, we unequivocally sexed 60 blastocysts, identifying 25 males and 35 females.

## Discussion

The recently published sequence of the cattle Y chromosome (Liu et al., 2019) has opened two avenues for the accurate sex determination of a sample from a subject that had not yet undergone gametogenesis. Each approach has its benefits and its limitations. For samples that are used for transcriptome analysis using RNA-sequencing and are limited in abundance, and researchers cannot extract both RNA and DNA, we have identified four genes present in the cattle chromosome Y that can be used for sex determination using transcriptome data alone. Although such analysis of transcriptome is not trivial, it can provide data on the sex of the sample, a biological variable that is often missing in many reports. On the other hand, for when researchers can obtain DNA, we produced and tested a set of oligonucleotides that allow an efficient determination of sex using PCR assays. This approach is very practical, and the data can be obtained quickly. Here we used these approaches successfully in cattle blastocysts.

### Using RNA-sequencing data to determine the sex of a sample

The mammalian chromosome Y are transcriptionally active during the pre-implantation and post-implantation development periods in tissues that are not directly related to the reproductive function (Chang et al., 2013; Petropoulos et al., 2016; Midic et al., 2018; Teixeira et al., 2019; Zhang et al., 2019; Godfrey et al., 2020). Our analysis focusing on the ampliconic region of the chromosome Y showed a greater abundance of transcripts from tissues found in gonads, but also detected transcripts in samples obtained from the liver, muscle, and prostate. We also observed samples from females that had sequences mapping to the ampliconic region of chromosome Y. This was not a surprising observation given the similarity between the DNA sequence in the chromosomes Y and X and the limitations of the mapping of short sequences (Ballouz et al., 2018; Webster et al., 2019).

Despite the similarity of DNA sequences between the chromosomes Y and X, we reasoned that specific genes would produce transcripts whose sequences would only map to the chromosomes Y in samples from male tissues. Indeed, four genes (ENSBIXG00000029788, ENSBIXG00000029892, ENSBIXG00000029774, and ENSBIXG00000029763) showed representative transcript abundance in male samples, with limited to no transcript detection in samples obtained from females. Only two out of four genes that can be used for determining sex in samples using RNA-sequencing data are annotated. The identifiers ENSBIXG00000029763 and ENSBIXG00000029774 are annotated to the genes “oral-facial-digit syndrome 1 centriole and centriolar satellite protein” and “zinc finger CCCH-type, RNA binding motif and serine/arginine rich 2,” respectively.

This pattern of transcription of Y-specific genes across tissues has not been observed in cattle before, but similar findings have been observed in other species for different genes. The genes *DDX3Y*, *KDM5D*, *ZFY*, *EIF2SEY*, and *EIF1AY* have been used for sexing of pig samples using RNA-sequencing data (Teixeira et al., 2019) while the genes *RPS4Y1* and *RPS4Y2* have been used for sexing of samples obtained from rhesus macaque (Midic et al., 2018) and the gene *Eif2s3y* has been used for sexing of mice samples using RNA-sequencing data (Midic et al., 2018). Thus, the selection of genes for sexing samples using RNA-sequencing data is different between species.

We expected that the identification of these candidate genes (ENSBIXG00000029788, ENSBIXG00000029892, ENSBIXG00000029774, and ENSBIXG00000029763) using a stringent systematic approach could be used to determine the sex of cattle samples which are yet undetermined. Our test in individual blastocysts indicated that these genes had a pattern of transcript abundance that resembled the observed in samples of known sex. Thus, we identified a set of genes that fit our initial hypothesis that RNA-sequencing data has valuable information that can be used for the accurate determination of sex in cattle embryos. Using our procedures, researchers can add sex as a variable in their investigation of the transcriptome using single embryos, in cases where there is no opportunity for DNA extraction for a PCR reaction. Even when using sexed semen to produce their embryos (Garner and Seidel, 2008; Sharpe and Evans, 2009), researchers can use the procedure presented here as a second-tier confirmation of the embryo’s sex. Although we did not test in this study, our results indicate that there is an opportunity to carry out embryo sexing using transcriptome data produced from a biopsy collected from a blastocyst.

### Using PCR to determine the sex of a sample

Many PCR assays have been developed for the determination of sex in samples, including from cattle (Herr, 1990; Itagaki et al., 1993; TAGLIAVINI et al., 1993; Fujishiro et al., 1995; Kamimura et al., 1997; Makondo et al., 1997; Zeleny et al., 2002; Seidel, 2003; Joerg et al., 2004; Parati et al., 2006; Abdul et al., 2011; Resende et al., 2011; Da Cruz et al., 2012; Davoudi et al., 2012; Herrero et al., 2013; Maleki et al., 2013), however, most of the assays have relied on oligonucleotides that anneal to repeated regions on the Y-chromosome (Bondioli et al., 1989; Utsumi et al., 1992; Itagaki et al., 1993; Machaty et al., 1993; Bredbacka et al., 1995; Park et al., 2001; Alves et al., 2003; Kageyama et al., 2004), or have limited efficiency (Kirkpatrick and Monson, 1993; Makondo et al., 1997; Hirayama et al., 2004; Zoheir and Allam, 2010; Abdul et al., 2011; Hommelsheim et al., 2014; Khamlor et al., 2015). The oligonucleotides we tested in this study have three important advantages. First, we used the complete sequence of the chromosome Y (Liu et al., 2019); second, we intentionally focused our search of candidate oligonucleotide pairs on the segment of the chromosome Y that is unique to males; third, we avoided repeated regions of the DNA. Our strategy to narrow down our search to a segment of the chromosome Y combined with an efficient tool for primer design [NCBI Primer BLAST (Ye et al., 2012)] allowed us to identify a set of oligonucleotides that produce a single amplicon that is of the same length for any sample originated from males. Although we tested the oligonucleotides in DNA from single blastocysts and used DNA from white blood cells for controls, this pair of oligonucleotides is expected to work with a wide range of tissues and initial amounts of DNA that is free from PCR inhibitors (Schrader et al., 2012).

In closing, the complete sequencing and annotation of genomes is essential for research in genomics and other disciplines. Working with the complete sequence of the cattle ChrY, we developed oligonucleotides that unequivocally determine the presence of the ChrY in a sample from cattle. A multiplex assay using oligonucleotides annealing to an autosome chromosome assures the assay efficiency in the absence of a chromosome Y in samples from females. We confirmed reports that the genes in the ChrY are transcriptionally active in tissues not directly related to reproductive functions, and such transcription is limited or inexistent for four genes in samples obtained from female cattle. This set of four genes can be used for the efficient and accurate sexing of samples using RNA-sequencing data in cattle.

## Data Availability

The data presented in the study are deposited in the NCBI-GEO repository, accession number GSE225693. Repository link: https://www.ncbi.nlm.nih.gov/geo/query/acc.cgi?acc=GSE225693.
