# Supplementary material for: Sexing of cattle embryos using RNA-sequencing data or polymerase chain reaction based on a complete sequence of cattle chromosome Y
Source: Front Genet. 2023 Apr 3;14:1038291. doi: 10.3389/fgene.2023.1038291 (PMC10106624; doi:10.3389/fgene.2023.1038291)
Supplement: Supplementary file 1 [file DataSheet1.PDF]

Supplementary code to: Sexing of cattle embryos using RNA-sequencing data or polymerase chain reaction based on the completed sequence of cattle chromosome Y

Jada Nix, Gustavo Schettini and Fernando Biase

2022-09-06

Table of Contents

Bioinformatics 1

- Alignment 1
- Filtering 2
- Counting 2

Analysis 2

- Libraries 2
- Obtain read counts 2
- Calculate differential expression 5
- Table 1 5
- Figure 1B 6
- Figure 1C 7

Code produced by Fernando Biase. I created this file to permit reproducibility of the findings described in the paper. Please direct questions to Fernando Biase: ***fbiase*** at ***vt.edu*** The sequence from the cattle chromosome Y published by Liu et al. 2019 can be obtained using this link CM011803.1 The gtf file with the annotation presented in Liu et al. 2019 is located here Bos_taurus_hybrid.UOA_Angus_1.97.gtf.gz

---

Bioinformatics

This is a general pipeline used for:

Alignment

```
#!/bin/bash
path_to_index=/home/fbiase/genome/arsucd_chrY/hisat_index/ars_ucd_1_2_chrY

/home/fbiase/bioinfo/hisat2-2.2.1/hisat2  -p 30 -k 1 -x $path_to_index -1 $read1  -2 $read2  -
-no-unal -S $output_alignment/alignment_chrY.sam  --summary-file $output_alignment/alignment_s
ummary.txt
```

Filtering

```
"converting sam to bam"

samtools sort  -O BAM -l 9 -o $folder/alignment_chrY.bam  $folder/alignment_chrY.sam  &

"indexing"
#####################################################

samtools index   $folder/alignment_chrY.bam   &

"filtering"
#####################################################

samtools view -b -h -F 1796 -o $folder/alignment_chrY.filtered.bam $folder/alignment_chrY.bam
&

"sorting"
#####################################################

samtools sort -O BAM -l 9 -o $folder/alignment_chrY.filtered.sorted.bam  $folder/alignment_chr
Y.filtered.bam &

"removing duplicates"
##################################################

bammarkduplicates I=$alignment O=$undup_output  level=9 markthreads=1 rmdup=1 index=1 dupindex
=0  verbose=0   &
```

Counting

```
gtf_file=/home/fbiase/genome/chrY/annotation/Bos_taurus_hybrid.UOA_Angus_1.97_only_chrY.chr.gt
f
/subread-2.0.1-source/bin/featureCounts -s 0 -a $gtf_file -o $output -F 'GTF' -t 'exon' -g 'ge
ne_id' --ignoreDup -T 30 -p  $undup_output
```

Analysis

Libraries

```
library('edgeR',quietly = TRUE, lib.loc="/usr/lib/R/site-library")
library("DESeq2",quietly = TRUE,lib.loc="/usr/lib/R/site-library")
library('dendextend', quietly = TRUE,lib.loc="/usr/lib/R/site-library")
library('ComplexHeatmap', quietly = TRUE,lib.loc="/usr/lib/R/site-library")
library('dendsort', quietly = TRUE,lib.loc="/usr/lib/R/site-library")
library('circlize', quietly = TRUE,lib.loc="/usr/lib/R/site-library")
library("ggplot2" ,quietly = TRUE, lib.loc="/usr/lib/R/site-library")
library("Rsamtools" ,quietly = TRUE, lib.loc="/usr/lib/R/site-library")
library("kableExtra" ,quietly = TRUE, lib.loc="/usr/lib/R/site-library")
library("flextable" ,quietly = TRUE, lib.loc="/usr/lib/R/site-library")
```

Obtain read counts

```
files<-list.files("/mnt/storage/lab_folder/chrY/alignment_chrY_public_data_a", recursive=T, pa
ttern="alignment_chrY.filtered.sorted.undup.bam", full.names = TRUE)
files<-files[grep("female", files, invert = FALSE)]
files<-files[grep("bai", files, invert = TRUE)]

index_stats_females<-data.frame(matrix(nrow=2213))
for (n in 1:length(files)) {
  index_stats<-Rsamtools::idxstatsBam(files[n])
  index_stats<-index_stats[,c(1,3)]
```

```
  colnames(index_stats)<-c("seqnames", stringr::str_split_fixed(files[n], "/", 9)[8])
  index_stats_females<-cbind(index_stats_females,index_stats)
}
index_stats_females<-subset(index_stats_females, select= -c(matrix.nrow...2213.))

#round( (index_stats_females[31,seq(from = 2, to = dim(index_stats_females)[2], by = 2)] /colS
ums(index_stats_females[,seq(from = 2, to = dim(index_stats_females)[2], by = 2)]) )*100 ,2)

dim(index_stats_females[,seq(from = 2, to = dim(index_stats_females)[2], by = 2)])[2]

## [1] 36

ave(colSums(index_stats_females[,seq(from = 2, to = dim(index_stats_females)[2], by = 2)]))[1]

## SRR1178419
##   17829956

min(round( (index_stats_females[31,seq(from = 2, to = dim(index_stats_females)[2], by = 2)] /c
olSums(index_stats_females[,seq(from = 2, to = dim(index_stats_females)[2], by = 2)]) )*100 ,2
))

## [1] 0.06

max(round( (index_stats_females[31,seq(from = 2, to = dim(index_stats_females)[2], by = 2)] /c
olSums(index_stats_females[,seq(from = 2, to = dim(index_stats_females)[2], by = 2)]) )*100 ,2
))

## [1] 0.16

rowMeans((index_stats_females[31,seq(from = 2, to = dim(index_stats_females)[2], by = 2)] /col
Sums(index_stats_females[,seq(from = 2, to = dim(index_stats_females)[2], by = 2)]) )*100 )

##         31
## 0.09959867

files<-list.files("/mnt/storage/lab_folder/chrY/alignment_chrY_public_data_a", recursive=T, pa
ttern="alignment_chrY.filtered.sorted.undup.bam", full.names = TRUE)
files<-files[grep("female", files, invert = TRUE)]
files<-files[grep("bai", files, invert = TRUE)]

index_stats_males<-data.frame(matrix(nrow=2213))
for (n in 1:length(files)) {
  index_stats<-Rsamtools::idxstatsBam(files[n])
  index_stats<-index_stats[,c(1,3)]
  colnames(index_stats)<-c("seqnames", stringr::str_split_fixed(files[n], "/", 9)[8])
  index_stats_males<-cbind(index_stats_males,index_stats)
}

index_stats_males<-subset(index_stats_males, select= -c(matrix.nrow...2213.))

#round( (index_stats_males[31,seq(from = 2, to = dim(index_stats_males)[2], by = 2)] /colSums(
index_stats_males[,seq(from = 2, to = dim(index_stats_males)[2], by = 2)]) )*100 ,2)

dim(index_stats_males[,seq(from = 2, to = dim(index_stats_males)[2], by = 2)])[2]

## [1] 30

ave(colSums(index_stats_males[,seq(from = 2, to = dim(index_stats_males)[2], by = 2)]))[1]

## SRR14740610
##    48700745
```

```
min(round( (index_stats_males[31,seq(from = 2, to = dim(index_stats_males)[2], by = 2)] /colSu
ms(index_stats_males[,seq(from = 2, to = dim(index_stats_males)[2], by = 2)]) )*100 ,2))

## [1] 0.05

max(round( (index_stats_males[31,seq(from = 2, to = dim(index_stats_males)[2], by = 2)] /colSu
ms(index_stats_males[,seq(from = 2, to = dim(index_stats_males)[2], by = 2)]) )*100 ,2))

## [1] 0.34

rowMeans((index_stats_males[31,seq(from = 2, to = dim(index_stats_males)[2], by = 2)] /colSums
(index_stats_males[,seq(from = 2, to = dim(index_stats_males)[2], by = 2)]) )*100 )

##        31
## 0.1382073

files<-list.files("/mnt/storage/lab_folder/chrY/alignment_chrY_public_data_a/male/counts_all",
recursive=T, pattern="count", full.names = TRUE)
files<-files[grep("summary", files, invert = TRUE)]
files<-files[grep(".sh", files, invert = TRUE)]
#length(files)
count_data_reference_males<-data.frame(matrix(nrow=27607))
for (n in 1:length(files)) {
  count<-read.delim(files[n], header=TRUE, sep= "\t", stringsAsFactors = FALSE, comment.char=
"#")
  count<-count[,c(1,7)]
  count_data_reference_males<-cbind(count_data_reference_males,count)
}
rownames(count_data_reference_males)<-count_data_reference_males[,2]
count_data_reference_males<-count_data_reference_males[,seq(from = 3, to = dim(count_data_refe
rence_males)[2], by = 2)]
colnames(count_data_reference_males)<- stringr::str_split_fixed(colnames(count_data_reference_
males), "[.]", 9)[,8]


files<-list.files("/mnt/storage/lab_folder/chrY/alignment_chrY_public_data_a/male/counts_chrY_
b", recursive=T, pattern="count", full.names = TRUE)
files<-files[grep("summary", files, invert = TRUE)]
files<-files[grep(".sh", files, invert = TRUE)]
#length(files)
count_data_chrY_males<-data.frame(matrix(nrow=192))
for (n in 1:length(files)) {
  count<-read.delim(files[n], header=TRUE, sep= "\t", stringsAsFactors = FALSE, comment.char=
"#")
  count<-count[,c(1,7)]
  count_data_chrY_males<-cbind(count_data_chrY_males,count)
}
rownames(count_data_chrY_males)<-count_data_chrY_males[,2]
count_data_chrY_males<-count_data_chrY_males[,seq(from = 3, to = dim(count_data_chrY_males)[2]
, by = 2)]
colnames(count_data_chrY_males)<- stringr::str_split_fixed(colnames(count_data_chrY_males), "[
.]", 9)[,8]

count_data_reference_chrY_males<-rbind(count_data_reference_males, count_data_chrY_males)

## [1] 119  64

## [1] 42 64
```

Calculate differential expression

```
group<-data.frame( row.names= c(colnames(count_data_reference_chrY_females), colnames(count_data_reference_chrY_males)),  sample= c(colnames(count_data_reference_chrY_females), colnames(count_data_reference_chrY_males)) , sex=rep(c("female", "male"), c(dim(count_data_reference_chrY_females)[2], dim(count_data_reference_chrY_males)[2])))

group$sex<-as.factor(group$sex)

design<-model.matrix(~  group$sex)

count_data_chrY_edger<-DGEList(count=count_data_reference_chrY_males_females)
count_data_chrY_edger<-estimateDisp(count_data_chrY_edger,design, robust=TRUE)

count_data_chrY_edger_QLFit <- glmQLFit(count_data_chrY_edger, design,robust=TRUE)
count_data_chrY_edger_QLFit <- glmQLFTest(count_data_chrY_edger_QLFit , coef="group$sexmale")
count_data_chrY_edger_QLFit_edgeR_results_QLF<- topTags(count_data_chrY_edger_QLFit, adjust.method = "fdr", n=Inf)$table

count_data_chrY_edger_QLFit_edgeR_results_QLF<-count_data_chrY_edger_QLFit_edgeR_results_QLF[with(count_data_chrY_edger_QLFit_edgeR_results_QLF, order(-F)),]

###

count_data_chrY_DeSeq2<-DESeqDataSetFromMatrix(countData=count_data_reference_chrY_males_females,colData=group, design= ~ sex)

# Wald method
count_data_chrY_DeSeq2_Wald<-DESeq(count_data_chrY_DeSeq2, test="Wald",fitType='local')
count_data_chrY_DeSeq2_Wald<-results(count_data_chrY_DeSeq2_Wald, contrast=c("sex",  "male", "female"), pAdjustMethod="fdr", tidy=TRUE)
count_data_chrY_DeSeq2_DESeq_Wald<-count_data_chrY_DeSeq2_Wald[with(count_data_chrY_DeSeq2_Wald, order(pvalue)),]

count_data_chrY_edgeR_DeSeq2<-merge(count_data_chrY_edger_QLFit_edgeR_results_QLF,count_data_chrY_DeSeq2_DESeq_Wald, by.x='row.names', by.y='row')

count_data_chrY_edgeR_DeSeq2<-count_data_chrY_edgeR_DeSeq2[with(count_data_chrY_edgeR_DeSeq2, order(-F)),]
```

Table 1

Table 1. Top ten differentially expressed genes between male and female tissues located in the cattle chromosome Y.

```
format_scientific <- function(x) {
  formatC(x, format = "e", digits = 2)
}

count_data_chrY_edger_QLFit_edgeR_results_QLF[1:10,]  %>% flextable::flextable() %>% set_formatter(PValue = format_scientific, FDR= format_scientific)  %>% colformat_double(j = c("logFC","logCPM","F"), digits = 2) %>% flextable::autofit()
```

logFC	logCPM	F	PValue	FDR
11.27	14.75	2,118.43	7.92e-37	1.11e-35
9.71	14.55	1,046.01	3.99e-46	1.68e-44
10.98	14.47	813.87	1.34e-28	9.37e-28
8.46	15.50	707.08	3.41e-40	7.16e-39
9.59	13.15	429.26	2.75e-23	1.65e-22
7.97	16.16	341.26	4.12e-29	4.32e-28
-1.95	18.42	320.06	6.13e-29	5.15e-28
6.60	15.14	228.70	5.33e-23	2.80e-22
6.73	15.57	210.27	4.26e-22	1.99e-21
-2.46	14.86	172.28	3.28e-21	1.38e-20

Figure 1B

```
col_fun = colorRamp2(c(min(cpm_data_reference_chrY_males_females), max(cpm_data_reference_chrY
_males_females)), c("#FFFFFF", "#1F305E"))

NCBI_GEO<-c("GSE55435", "GSE55435", "GSE55435", "GSE55435", "GSE55435", "GSE55435", "GSE55435"
, "GSE55435", "GSE55435", "GSE55435", "GSE55435", "GSE55435", "GSE55435", "GSE55435",  "GSE554
35", "GSE55435", "GSE55435", "GSE55435", "GSE55435", "GSE55435" , "GSE55435", "GSE55435","GSE5
5435", "GSE55435", "GSE55435", "GSE55435", "GSE55435", "GSE55435" , "GSE55435", "GSE55435", "G
SE55435", "GSE192530", "GSE192530", "GSE192530", "GSE192530", "GSE192530","GSE176219", "GSE176
219", "GSE176219", "GSE176219", "GSE176219", "GSE176219", "GSE176219", "GSE176219", "GSE196974
", "GSE196974", "GSE196974", "GSE196974", "GSE196974", "GSE196974", "GSE196974", "GSE196974",
"GSE196974", "GSE196974", "GSE196974", "GSE196974", "GSE196974", "GSE196974", "GSE196974", "GS
E196974", "GSE128075", "GSE128075", "GSE128075",  "GSE128075")

samples<-c("hypothalamus_1", "hypothalamus_2", "hypothalamus_3", "hypothalamus_4","pituitary_1
", "pituitary_2", "pituitary_3", "pituitary_4", "ovary_1", "ovary_2", "ovary_3", "ovary_4", "u
terus_1", "uterus_2", "uterus_3", "uterus_4", "endometrium_1", "endometrium_2", "endometrium_3
", "muscle_1", "muscle_2", "muscle_3", "muscle_4", "fat_1" , "fat_2" , "fat_3" , "fat_4" , "li
ver_1", "liver_2", "liver_3", "liver_4", "PWBC_1", "PWBC_2", "PWBC_3", "PWBC_4", "PWBC_5",  "t
estis_1", "testis_2", "testis_3", "testis_4", "testis_5", "testis_6", "testis_7", "testis_8",
"muscle_1", "muscle_2", "muscle_3", "muscle_4", "muscle_5", "muscle_6", "muscle_7", "muscle_8"
, "liver_1", "liver_2", "liver_3", "liver_4", "liver_5", "liver_6", "liver_7", "liver_8", "pro
state_1", "epididymis_1", "epididymis_2", "testis_2")


#colnames(cpm_data_reference_chrY_males_females)<- paste(samples,NCBI_GEO)

colnames(cpm_data_reference_chrY_males_females)<- paste(samples)


#colnames(cpm_data_reference_chrY_males_females)



Heatmap(cpm_data_reference_chrY_males_females,
                name= "CPM",
               cluster_rows = dendsort(fastcluster::hclust(dist(cpm_data_reference_chrY_males_
females), method = "single")),
```

```
                 cluster_columns = dendsort(fastcluster::hclust(dist(t(cpm_data_reference_chrY_m
ales_females)), method = "single")),
                 show_row_names = TRUE,
                 col = col_fun,
                 show_heatmap_legend = TRUE,
                 top_annotation = HeatmapAnnotation(
                             "sex"= factor(rep(c("female", "male"), c(dim(count_data_reference_ch
rY_females)[2], dim(count_data_reference_chrY_males)[2]))), "dataset"= factor(NCBI_GEO) , whic
h = c("column"), col=list("sex"=setNames(c("#cc79a7","#56b3e9"), c("female","male")),"dataset"
=setNames(c("#c65b80", "#77a451", "#9765ca", "#c7733b", "#6c93c5"),c("GSE55435", "GSE176219",
"GSE196974", "GSE128075", "GSE192530"))) , show_annotation_name = FALSE, show_legend = TRUE))
```

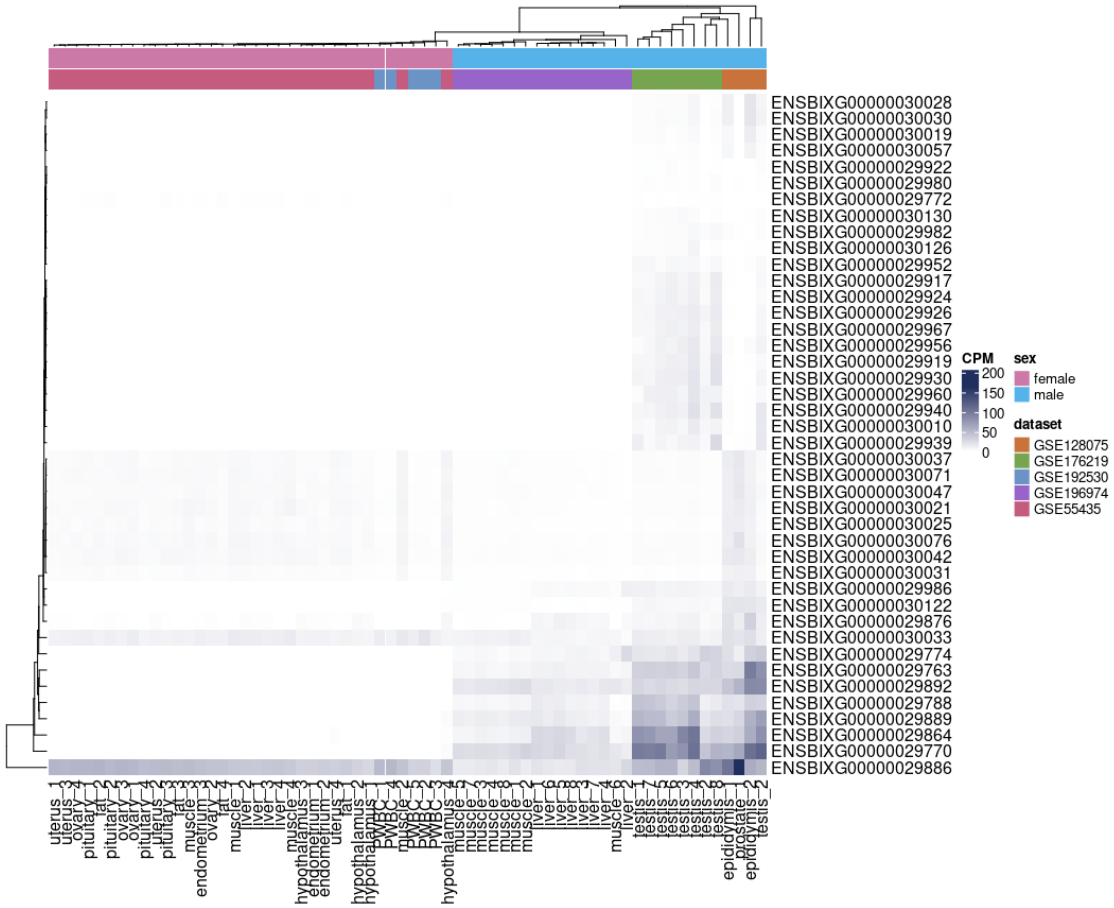

Figure 1C

```
colnames(cpm_data_reference_chrY_males_females)<- paste(samples,NCBI_GEO)

cpm_data_reference_chrY_males_females<-as.data.frame(cpm_data_reference_chrY_males_females)
cpm_data_reference_chrY_males_females_graph<-cpm_data_reference_chrY_males_females[rownames(cp
m_data_reference_chrY_males_females) %in% c("ENSBIXG00000029788","ENSBIXG00000029892","ENSBIXG
00000029774", "ENSBIXG00000029763"),]

cpm_data_reference_chrY_males_females_graph$gene<-rownames(cpm_data_reference_chrY_males_femal
es_graph)
cpm_data_reference_chrY_males_females_graph<-reshape::melt(cpm_data_reference_chrY_males_femal
es_graph,id.vars="gene")

ggplot(cpm_data_reference_chrY_males_females_graph, aes(x=reorder(variable, value, sum), y=val
ue, fill=gene )) +
```

```
    geom_bar(position="stack", stat="identity")+
    #geom_hline(yintercept=500, linetype="dashed", color = "red")+
    scale_y_continuous(name="Counts per million reads")+
    scale_x_discrete(name=NULL)+
    scale_fill_manual(values=c("#015fe3","#c57b00","#8a2f6f","#00a56a","#ffaae3","#ff8060"))+
    #facet_grid(~sex,scales="free")+
    theme_classic()+
    theme(
    axis.title  = element_text(size=15, color="black"),
    axis.text.x = element_text(size=13, color="black", angle=90,vjust=0.5, hjust=1),
    axis.text.y = element_text(size=15, color="black"),
    legend.text = element_text(size=15, color="black"),
    legend.title = element_text(size=15, color="black"),
    legend.position = c(0.2, 0.8)
)
```

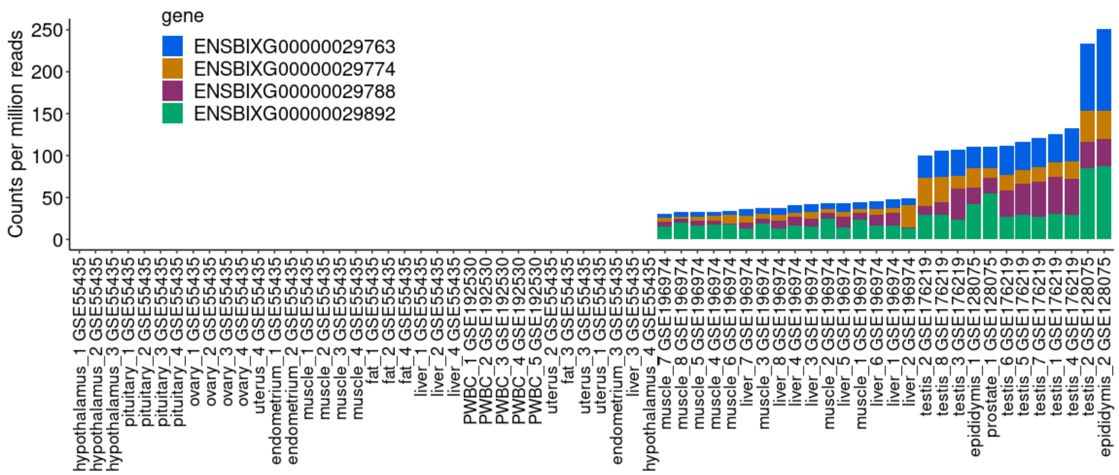


```
cpm_data_reference_chrY_males_females<-as.data.frame(cpm_data_reference_chrY_males_females)
cpm_data_reference_chrY_males_females_graph<-cpm_data_reference_chrY_males_females[rownames(cp
m_data_reference_chrY_males_females) %in% c("ENSBIXG00000029788","ENSBIXG00000029892","ENSBIXG
00000029774", "ENSBIXG00000029763"),]

summary(colSums(cpm_data_reference_chrY_males_females_graph[,1:dim(count_data_reference_chrY_f
emales)[2]]))

##    Min. 1st Qu.  Median    Mean 3rd Qu.    Max.
## 0.00000 0.00000 0.00000 0.03041 0.00000 0.36775

summary(colSums(cpm_data_reference_chrY_males_females_graph[,37:dim(cpm_data_reference_chrY_ma
les_females_graph)[2]]))

##    Min. 1st Qu.  Median    Mean 3rd Qu.    Max.
##   30.92   37.94   46.76   80.65  111.24  250.85

## [1] 119  22
```

##Figure 1B

```
cpm_data_reference_chrY_blastocysts<-as.data.frame(cpm_data_reference_chrY_blastocysts)
cpm_data_reference_chrY_blastocysts_graph<-cpm_data_reference_chrY_blastocysts[rownames(cpm_da
ta_reference_chrY_blastocysts) %in% c("ENSBIXG00000029788","ENSBIXG00000029892","ENSBIXG000000
29774", "ENSBIXG00000029763"),]

cpm_data_reference_chrY_blastocysts_graph$gene<-rownames(cpm_data_reference_chrY_blastocysts_g
raph)
```

```
cpm_data_reference_chrY_blastocysts_graph<-reshape::melt(cpm_data_reference_chrY_blastocysts_g
raph,id.vars="gene")



ggplot(cpm_data_reference_chrY_blastocysts_graph, aes(x=reorder(variable, value, sum), y=value
, fill=gene )) +
    geom_bar(position="stack", stat="identity")+
    #geom_hline(yintercept=500, linetype="dashed", color = "red")+
    scale_y_continuous(name="Counts per million reads")+
    scale_x_discrete(name=NULL)+
    scale_fill_manual(values=c("#015fe3","#c57b00","#8a2f6f","#00a56a","#ffaae3","#ff8060"))+
    #facet_grid(~sex,scales="free")+
    theme_classic()+
    theme(
    axis.title  = element_text(size=15, color="black"),
    axis.text.x = element_text(size=13, color="black", angle=90,vjust=0.5, hjust=1),
    axis.text.y = element_text(size=15, color="black"),
    legend.text = element_text(size=15, color="black"),
    legend.title = element_text(size=15, color="black"),
    legend.position = c(0.2, 0.8)
)
```

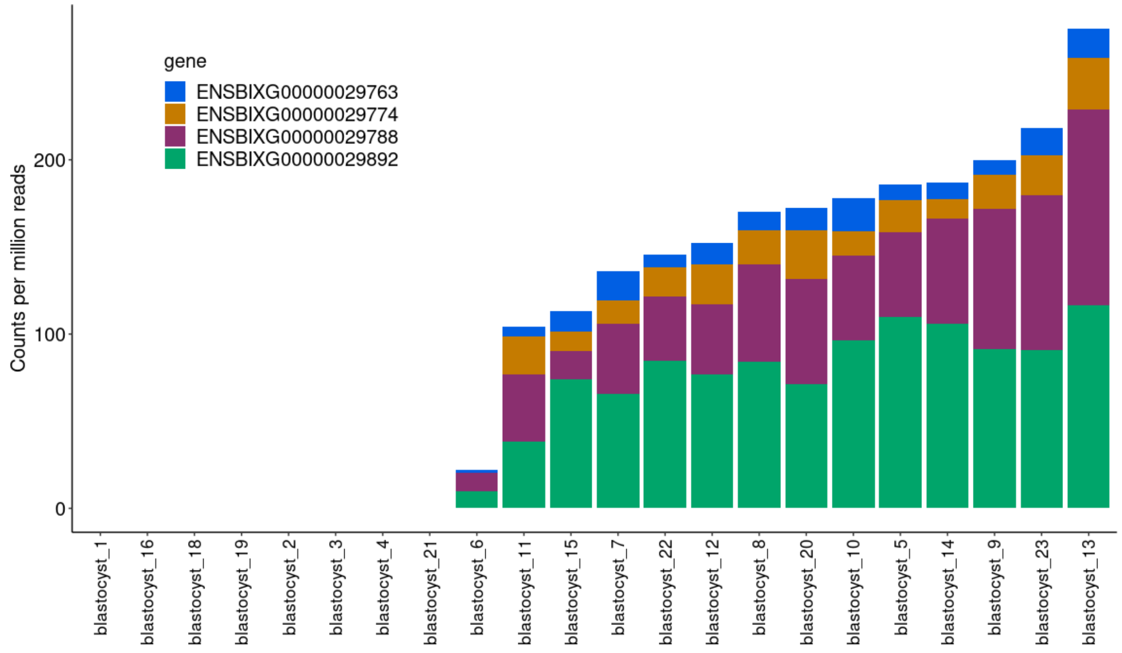


```
cpm_data_reference_chrY_blastocysts<-as.data.frame(cpm_data_reference_chrY_blastocysts)
cpm_data_reference_chrY_blastocysts_graph<-cpm_data_reference_chrY_blastocysts[rownames(cpm_da
ta_reference_chrY_blastocysts) %in% c("ENSBIXG00000029788","ENSBIXG00000029892","ENSBIXG000000
29774", "ENSBIXG00000029763"),]

cpm_data_reference_chrY_blastocysts_graph

##                    blastocyst_1 blastocyst_10 blastocyst_11 blastocyst_12 blastocyst_13 bla
stocyst_14 blastocyst_15 blastocyst_16 blastocyst_18 blastocyst_19 blastocyst_2 blastocyst_20
blastocyst_21 blastocyst_22 blastocyst_23 blastocyst_3 blastocyst_4 blastocyst_5 blastocyst_6
blastocyst_7 blastocyst_8 blastocyst_9
## ENSBIXG00000029763            0      18.97552      6.061083      11.92225      16.87044
9.758044      11.71454             0             0             0            0      13.06061
0.00000000      7.238592      15.68339            0            0     9.008661    1.5926710
```

```
16.49501     10.23573     8.045926
## ENSBIXG00000029774            0      13.81169     21.483837      22.84052      29.46628
11.051928      10.75545             0             0             0             0      27.95786
0.00000000     16.930944      22.96496             0             0    18.017322    0.2654452
13.44391     19.97079    19.674804
## ENSBIXG00000029788            0      48.65200     38.826934      40.59839     112.22264
60.057797      16.64698             0             0             0             0      60.26926
0.00000000     36.745056      89.00830             0             0    48.617393   10.6178064
40.28406     55.57332    80.459261
## ENSBIXG00000029892            0      96.30856     38.106806      76.74163     116.72522
106.098513      73.84956             0             0             0             0      71.35717
0.05925871     84.593375      90.53591             0             0   110.062337    9.5560257
65.74168     84.22223    91.522410

colSums(cpm_data_reference_chrY_blastocysts_graph)

##  blastocyst_1 blastocyst_10 blastocyst_11 blastocyst_12 blastocyst_13 blastocyst_14 blastoc
yst_15 blastocyst_16 blastocyst_18 blastocyst_19  blastocyst_2 blastocyst_20 blastocyst_21 bla
stocyst_22 blastocyst_23  blastocyst_3  blastocyst_4  blastocyst_5  blastocyst_6  blastocyst_7
blastocyst_8  blastocyst_9
##    0.00000000  177.74778193  104.47865989  152.10279287  275.28457472  186.96628283  112.96
652985    0.00000000    0.00000000    0.00000000    0.00000000  172.64489876    0.05925871  14
5.50796604  218.19255399    0.00000000    0.00000000  185.70571280   22.03194818  135.96466597
170.00207663  199.70240131
```
